# Validated HPLC method for simultaneous determination of azelastine hydrochloride fluticasone propionate and oxymetazoline in nasal mucosa and nasopharyngeal swabs from real human samples

**DOI:** 10.1038/s41598-024-82387-7

**Published:** 2025-02-04

**Authors:** Neven N. Mikawy, Nancy Magdy, Marwa H. Mohamed, Amira M. El-Kosasy

**Affiliations:** Department of Pharmaceutical Analytical Chemistry, Faculty of Pharmacy, Ain Shams University, Abbassia, Cairo, 11566 Egypt

**Keywords:** Nasopharyngeal swabs, Nasal mucosa, Seasonal allergy, HPLC, Azelastine hydrochloride, Fluticasone propionate, Oxymetazoline, Clinical trial design, Biochemistry, Biological techniques

## Abstract

A combination of three co-administrated drugs, such as azelastine hydrochloride (AZT), fluticasone propionate (FP), and oxymetazoline (OXY), is more effective than single therapy for the treatment of seasonal allergy and COVID-19. We established an efficient methodology for the determination of those analytes in spiked nasal mucosa and nasopharyngeal swabs from real human samples. A simple and quick protein precipitation method was used for sample extraction, using acetonitrile. RP-HPLC/DAD method was performed using an Exsil 100 ODS C18 (250 × 4.6 mm, 5 μm) column with an acetonitrile: water (70:30 v/v) solvent system at a flow rate of 0.7 mL/min. A photodiode array detector was applied at 240 nm. A good separation of the three proposed analytes with a short run time of 10 min was noted. Our method was validated according to FDA guidelines for bioanalytical validation methods. Calibration curves were linear in nasal mucosa samples at concentration ranges of 8–125, 10–100, and 10–125 µg/mL, with average recoveries ± SD of 101.56%±0.39, 102.45%±0.86, and 104.61%±4.52 for AZT, FP, and OXY; respectively. The results of precision and accuracy are within acceptable limits. According to stability assays, the three analytes under investigation were stable throughout sample preparation, storage, and injection. Our method was applied to real nasopharyngeal swabs. It shows that the results of the swabs were not affected by gender or age. Good recoveries with low % RSD were observed: 99.03% ± 0.75, 100.02% ± 0.94, and 100.94% ± 1.98 for both genders, and 100.45% ± 0.96, 100.69% ± 1.08, and 100.32% ± 1.53 for different ages for AZT, FP, and OXY; respectively. Moreover, the amount of those drugs in the nasal mucosa was observed for seven hours, and a constant concentration with a low% RSD was noted for the first four hours. Therefore, this method can be applied to monitor the therapeutic dose in the nasal mucosa for the determination of those analytes.

## Introduction

Allergy rhinitis is an allergy-induced symptomatic condition of the nose caused by inflammation mediated by immunoglobulin E (IgE) following exposure of the nasal membranes to allergens. In reaction to the allergen, IgE antibodies are generated, and upon adhering to mast cells, they release inflammatory mediators such as histamine^1^. The most common triggers are molds and pollens^2^. During the disease’s progression, a patient may experience one or more of the following symptoms, which must last for at least two days and at least an hour each day. These symptoms can be resolved on their own or with medical intervention. The symptoms include nasal blockage, sneezing, nasal discharge, and nasal irritation^3,4^. Pharmacotherapy, allergen immunotherapy, and avoiding allergens and irritants have long been part of the treatment algorithm for patients with seasonal allergy rhinitis (SAR)^5^. SAR pharmacotherapy is based on many drug classes, which can be used as monotherapy in some circumstances or as part of mixed regimens. These regimens include intranasal steroids, decongestants, leukotriene inhibitors, mast cell stabilizers, oral and intranasal antihistamines, and intranasal anticholinergics. Combining azelastine (AZT) with fluticasone propionate (FP) in one intranasal device has been shown to be more clinically successful^6^.

SARS-CoV-2 causes the complicated clinical syndrome known as coronavirus disease 2019 (COVID-19)^7^. Since the nose and nasopharynx often have the highest viral loads of Coronavirus 2 (SARS-CoV-2), utilizing nasal spray reduces the virus’s load. Coughing, fever, rhinitis, and loss of taste and smell are considered the most common early signs of SARS-CoV-2 infection^8^.

**Fig. 1:**
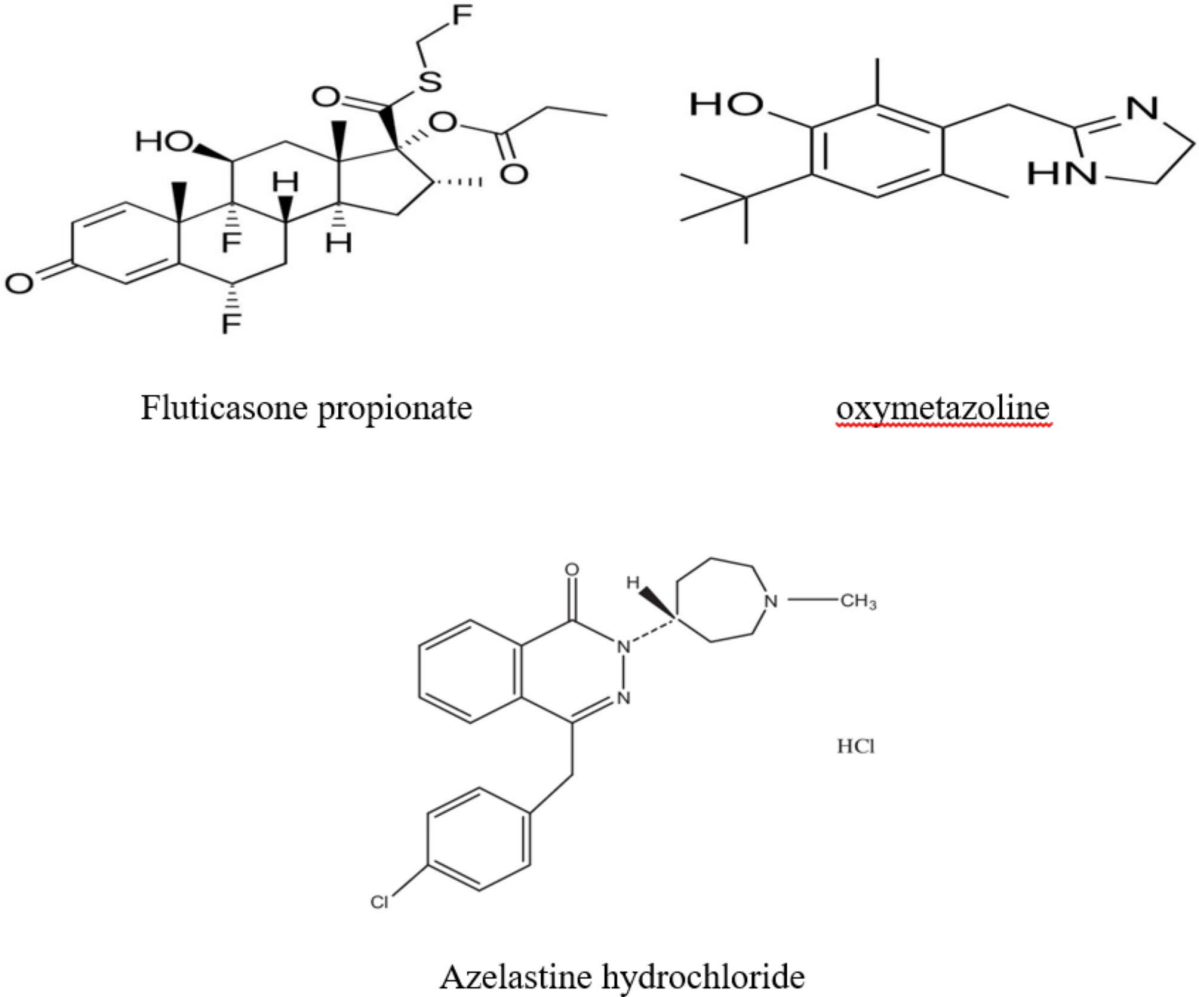
Structure of seasonal allergy drugs under study.

Azelastine hydrochloride; 4-(4-chlorobenzyl)-2-(1-methylazepan-4-yl) phthalazin-1(2 H)-one (Fig. 1)^9^ was approved in September 2000 as a drug used for the treatment of SAR^10^. It contains anti-inflammatory and mast-cell stabilizing qualities, which reduce the levels of leukotrienes, kinins, and platelet activating factors both in vivo and in vitro. With its quick start of action, azelastine nasal spray relieves rhinitis-related nasal symptoms such as post-nasal drip and congestion^11^. Also, azelastine hydrochloride is used in COVID-19 patients as it works well to hasten the decrease of the virus load in the nasal cavity and alleviate the general symptoms that COVID-19 patients have observed^12^.

Fluticasone propionate (FP) [(6 S,8 S,9R,10 S,11 S,13 S,14 S,16R,17R)-6,9-difluoro-17-(fluoromethylsulfanylcarbonyl)-11-hydroxy-10,13,16-trimethyl-3-oxo-6,7,8,11,12,14,15,16-octahydrocyclopenta[a]phenanthren-17-yl] propanoate^13^ was launched in 1999 as a drug used for the treatment of SAR (Fig. 1)^14^. FP is a glucocorticoids (GR) drug that acts as an anti-inflammatory drug by different mechanisms. Such mechanisms accelerate the production of anti-inflammatory genes^15^. Furthermore, FP’s propionate ester side chain makes it extremely lipophilic, which enables it to bind tissue more quickly and firmly and to retain its bond longer than more hydrophilic compounds like hydrocortisone and budesonide^16^. COVID-19 patients who received fluticasone nasal spray showed a significant improvement in smell and taste within a week^17^.

Oxymetazoline (OXY) 6-tert-butyl-3-(4,5-dihydro-1 H-imidazol-2-ylmethyl)-2,4-dimethylphenol is an Adreno mimetic that, when administered topically, non-selectively agonizes α1 and α2-adrenergic receptors as well as endothelial postsynaptic α2 receptors, causing vasoconstriction in nasal vascular beds (Fig. 1)^18^. Such vasoconstriction increases the airway lumen’s diameter, which relieves nasal congestion^19^. Oxymetazoline nasal drops are suggested to be taken to decrease transportation of COVID-19 between health care workers^20^.

As far as we are aware, there are no documented HPLC and LC-MS-MS procedures reports for the concurrent assessment of the three co-administered investigated medications (FP, OXY, and AZT) in the nasal mucosa. An HPLC-UV method was reported for the determination of AZT in nasal spray formulations^21^. Different HPLC-UV detector methods were reported for the determination of AZT and FP in dosage form^22–24^. And only one TLC method was applied for the determination of AZT and FP in nasal spray dosage form^25^. Other methods were stated for the separation of AZT and FP^26,27^.

This work suggested an efficient, simple, and fast RP-HPLC technique for simultaneous measurement of FP, OXY, and AZT in nasal mucosa. Moreover, HPLC instruments are less expensive and more readily available than LC–MS–MS, therefore they are more suitable for widespread everyday use, especially in poor nations. As a result, the suggested approach aids in the assessment of medication effectiveness, optimization of treatment, and quantification of drugs in the nasal mucosa.

## Experimental

### Materials and reagents

Azelastine hydrochloride (AZT), Fluticasone propionate (FP) and Oxymetazoline (OXY) standards were provided by Amoun Pharmaceutical Company (Obour city, Cairo, Egypt), with purity 99.90%. All reagents were of HPLC grade. Methanol, acetonitrile, and water were obtained from Cornal Lab (Cairo, Egypt). Nasal mucosa was collected from healthy volunteers.

### Dosage forms

Azelast plus nasal spray^®^ product contains 125 µg of azelastine hydrochloride and 50 µg of fluticasone propionate in each 0.137 mL.

Oxymet nasal drop^®^ product contains 0.50 mg of oxymetazoline HCl in each 1 mL.

### Instrumentation and HPLC conditions

Chromatographic analysis of the pharmaceuticals under investigation was conducted using the Hitachi Chromaster HPLC-DAD-FL system from VWR, which is outfitted with an autoinjector, quaternary pump, vacuum degasser, Hitachi Chromaster 5430 diode array detector, and Hitachi Chromaster 5440 florescence detector (Japan). The stationary phase was an Exsil 100 ODS C18 column (250 × 4.6 mm, 5 μm) from Dr Maisch GmbH (Ammerbuch, Germany), while the mobile phase was a mixture of 30:70 v/v acetonitrile and water. All solvents were filtered and degassed using a 0.40 μm membrane filter before use. The run time was set at 10 min at a flow rate of 0.70 mL/min and UV detection at 450 nm. Between runs, the mobile phase was used for equilibration and conditioning.

### Procedure

#### Preparation of standard calibration curve and quality control samples

Stock solutions (100 µg/mL) of azelastine hydrochloride (AZT), fluticasone propionate (FP), and oxymetazoline (OXY) were prepared by dissolving 0.01 gm of each standard in a 100 mL volumetric flask and completing the final volume using methanol, then stored at 4 °C.

For the preparation of working solutions, accurate volumes from the stocks were applied to 900 µL of nasal mucosa to prepare final concentrations of (8, 15, 35, 50, 100, 125) µg/mL for AZT, (10, 25, 35, 50, 75, 100) µg/mL for FP, and (10, 25, 35, 50, 75, 125) µg/mL for OXY as calibrator samples.

Quality control samples (QCs) were prepared by diluting accurate volume from the stocks to 900 µl of nasal mucosa to prepare final concentrations of (8.00, 24.00, 62.50, 87.50) µg/mL for AZT, (10, 30, 50, 70) µg/mL for FP, and (10.00, 30.00, 62.50, 87.50 µg/mL for OXY as lower limit of quantification (LLOQ), low QC (LQC), mid QC (MQC), and high (HQC); respectively.

#### Sample preparation

A straightforward protein precipitation method was employed: 1 mL of spiked nasal mucosa was added to 2 mL of acetonitrile, then centrifuged for 10 min at 6,000 rpm. Supernatant was collected and passed through a 0.45 μm filter, prior to injection. Among the various solvents and ratios tested in the extraction process, such as methanol, methanol: acetonitrile, acetonitrile. Finally, acetonitrile demonstrated the highest extraction power with acceptable system suitability parameters.

### Method validation

In the validation of the method, the US Food and Drug Administration’s guidelines (FDA) for the validation of bio-analytical methods were followed^28^.

### Selectivity

Selectivity was applied to ensure that there was no interference between the matrix (nasal mucosa) and the analytes of interest. Blank nasal mucosa was taken from different sources and was compared with nasal mucosa spiked with LLOQ of each analyte.

### Calibration curve and linearity

Nasal mucosa was accurately spiked with AZT, FP, and OXY to obtain concentration ranges of 8–125 µg/mL, 10–100 µg/mL, and 10–125 µg/mL for each analyte, respectively. Each sample was prepared and then injected into HPLC under the previously stated chromatographic conditions. The average peak areas for every sample were then determined. Each analyte’s observed peak areas were plotted against their matching concentration, and the regression equation was subsequently determined.

### Accuracy and precision

Evaluating accuracy and precision is very crucial to ensure that our method is ready for analysis of proposed drugs. Our method was applied to four levels of QCs (LLOQ, LOQ, MQC, and HQC) on the same day for intraday accuracy and on three successive days for inter-day accuracy. Each QC was determined five times for each analyte. It was expressed as a mean recovery % ± SD and precision as %RSD.

### Stability

Stability was determined at two levels of QCs (LQC and HQC) for each analyte. Each sample was determined three times, and then the average recovery was calculated. For bench-top stability, it was evaluated by leaving spiked nasal mucosa samples at room temperature for 8 h. For freeze-thaw stability, nasal mucosa was frozen for 12 h, then thawed and used for the preparation of QCs. This procedure was applied at least three times. Finally, for autosampler stability, spiked nasal mucosa samples were left at the autosampler 24 h.

### Dilution integrity

Dilution integrity was calculated *via* spiking the nasal mucosa matrix with the three drugs at a high concentration level above ULOQ, then diluting it with blank to reach the assay range. Twelve samples were prepared, six were diluted twice, and the remaining were diluted four times to reach the assay range. The proposed method was conducted to analyze the samples.

### Robustness

Robustness quantifies the method’s capacity to be constant with slight, deliberate changes in method parameters. It was assessed by determining the %RSD following a modification to the mobile phase ratio, acetonitrile: water from (70:30) to (68:32) and (72:28). Also, the flow rate of the mobile phase was slightly changed by (± 0.10 mL/min).

### Matrix effect (ME)

It was applied to describe the reaction between samples and components that were found in the matrix. It was done at three levels of QCs (LQC, MQC and HQC) for each analyte, after which the peak area of spiked samples was compared with that of pure samples and represented as %ME. If the % ME is greater than 100%, matrix components have an enhancement effect and vice versa.

### Application to spiked nasal mucosa and real samples

For spiked nasal mucosa, 1 mL was spiked with three QCS (LQC, MQC, and HQC) of each analyte, and then samples were prepared as the procedure mentioned before. After the preparation of samples, they were filtrated and injected into the HPLC system. The mean recovery% ± SD was calculated.

For real samples, the study was approved by the Ethics Committee at the Faculty of Pharmacy Ain Shams University (approval number, REC#273). The method was carried out in accordance with relevant guidelines and informed consent was obtained from all subjects. The previously described method was applied for the determination of three proposed analytes in nasopharyngeal swabs after taking one dose of azelast plus nasal spray and an oxymet drop in the nose. The nasopharyngeal swabs were first applied to healthy volunteers, male and female of different ages. After comparing peak areas of nasopharyngeal swabs from both genders, the same drugs were given to people of different ages (20, 40, and 60 years old) to know if the results of the nasopharyngeal swabs would be affected by age. Finally, the nasopharyngeal swabs were taken at different intervals. Swabs were transferred to a falcon tube containing 1 ml of acetonitrile, and sample preparation was continued as mentioned before and injected into the HPLC system to determine the concentration of each analyte in different swabs.

## Results and discussion

**Fig. 2:**
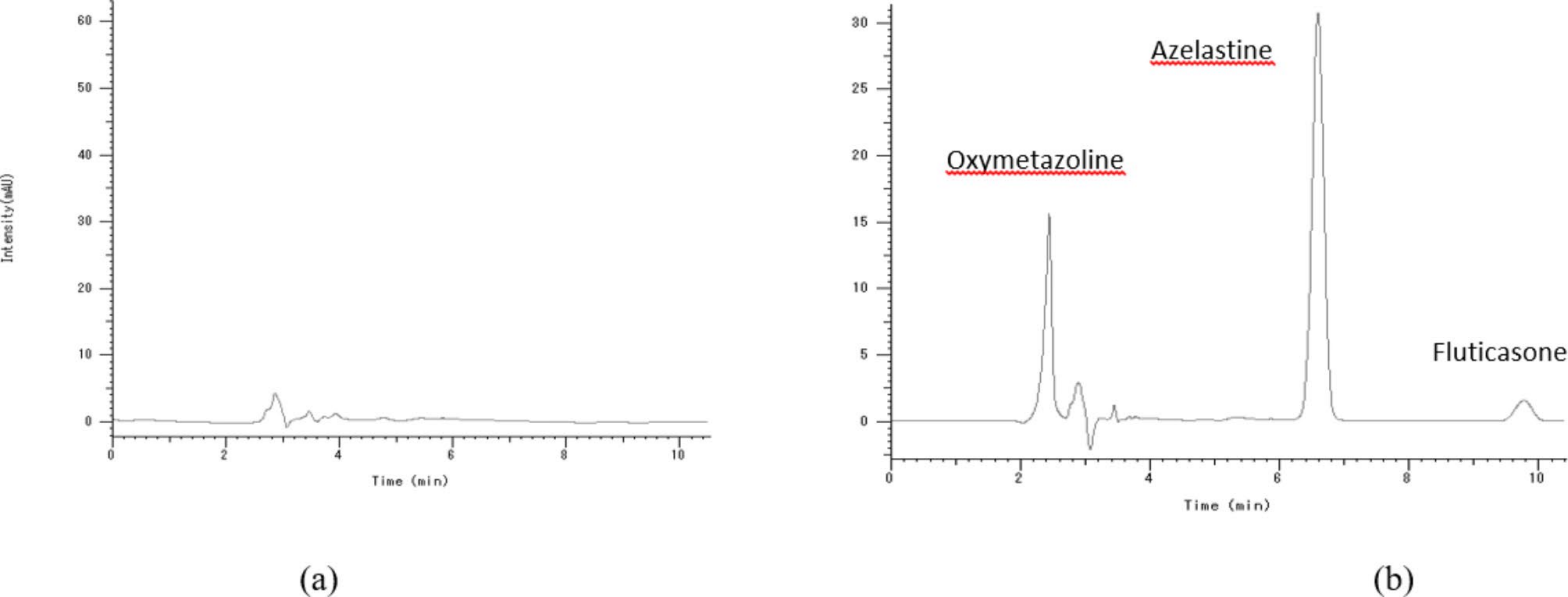
HPLC chromatograms of (**a**) Blank nasal mucosa, (**b**) nasal mucosa spiked with 50 µg/mL of AZT (RT 6.61 min.), FP (RT 9.81 min.), and OXY (RT 2.45 min.).

Our method shows good selectivity, with no peaks observed in the blank nasal mucosa chromatogram, at the retention time of each analyte (Fig. 2.**a**). Three proposed analytes were separated and determined quantitatively in the nasal mucosa with our developed method, as shown in (Fig. 2.**b**).

The FDA standards were followed in the bio analytical validation of the method, which included the validation criteria of selectivity, linearity, accuracy, precision, stability, robustness, and matrix effect.

### Linearity and sensitivity

The calibration curves of the three proposed analytes are linear within the range of (8-125), (10–100), and (10–125) µg/mL for AZT, FP, and OXY, respectively. The calibration curve for each analyte was plotted, while the regression equation and slope were mentioned in (Table 1).

**Table 1:** Linearity data of the proposed HPLC method for the determination of AZT, FP and OXY in nasal mucosa.

Parameter	AZT	FP	OXY
Concentration range (µg/mL)	8-125	10–100	10–125
*Linearity*			
Intercept	+ 231,591	− 12,629	+ 50,350
Slope	12,506	1561.7	7384.5
Correlation coefficient (r)	0.9994	0.9993	0.9994
LLOQ (µg/ml)	8.00	10.00	10.00

The sensitivity of our method was calculated through the determination of the LLOQ of each analyte. LLOQ was found to be 8 µg/mL for AZT and 10 µg/mL for both FP and OXY (Table 1).

### Accuracy and precision

The results displayed in (Table 2**)** showed low SD and low % RSD, which demonstrated the accuracy and precision of the suggested method, which enabled us to determine the proposed analytes in the nasal mucosa.

**Table 2:** Accuracy and precision of the proposed HPLC method for the determination of AZT, FP and OXY in nasal mucosa.

Samples	AZT		FP		OXY	
	Conc (µg/mL)	Recovery%	Conc (µg/mL)	Recovery%	Conc (µg/mL)	Recovery%
LLOQ	8.00	103.95	10.00	103.67	10.00	103.66
LQC	24.00	102.59	30.00	98.60	30.00	106.07
MQC	62.50	101.83	50.00	99.23	62.50	99.89
HQC	87.50	98.40	70.00	101.07	87.50	102.35
Mean ± SD	101.69 ± 2.36		100.64 ± 2.28		102.99 ± 2.58	

Samples	Intraday precision					
	AZT		FP		OXY	
	Conc (µg/mL)	Mean recovery*% ±RSD	Conc (µg/mL)	Mean recovery*% ±RSD	Conc (µg/mL)	Mean recovery*% ±RSD
LLOQ	8.00	106.80 ± 3.83	10.00	107.67 ± 0.89	10.00	103.66 ± 8.34
LQC	24.00	102.10 ± 0.46	30.00	98.05 ± 1.20	30.00	107.78 ± 5.98
MQC	62.50	101.78 ± 0.09	50.00	98.88 ± 0.71	62.50	97.60 ± 5.52
HQC	87.50	99.08 ± 0.98	70.00	100.75 ± 0.42	87.50	100.62 ± 2.02

Samples	Inter-day precision					
	AZT		FP		OXY	
	Conc (µg/mL)	Mean recovery*% ±RSD	Conc (µg/mL)	Mean recovery*% ±RSD	Conc (µg/mL)	Mean recovery*% ±RSD
LLOQ	8.00	104.63 ± 5.57	10.00	106.30 ± 0.53	10.00	111.73 ± 7.63
LQC	24.00	101.20 ± 1.41	30.00	102.76 ± 1.08	30.00	107.09 ± 9.86
MQC	62.50	101.17 ± 0.07	50.00	98.96 ± 2.73	62.50	94.97 ± 6.25
HQC	87.50	101.28 ± 0.18	70.00	99.67 ± 4.60	87.50	98.53 ± 3.71

*Average of three determinations.

### Stability

Stability of the Analytes in the nasal mucosa was evaluated using bench-top, freeze-thaw, and autosampler stability. The results obtained showed good stability (Table 3).

**Table 3:** Stability results of AZT, FP and OXY in nasal mucosa under different stability assessment conditions.

Samples	AZT		FP		OXY	
	Conc (µg/mL)	Mean recovery*% ± RSD	Conc (µg/mL)	Mean recovery*% ±RSD	Conc (µg/mL)	Mean recovery *% ±RSD
Bench top stability						
LQC	24.00	98.16 ± 0.50	30.00	98.72 ± 2.17	30.00	96.90 ± 1.95
HQC	87.50	99.91 ± 0.93	70.00	102.66 ± 0.92	87.50	102.45 ± 2.04
Auto-sampler stability						
LQC	24.00	99.98 ± 1.75	30.00	103.23 ± 2.91	30.00	98.61 ± 0.38
HQC	87.50	101.25 ± 0.84	70.00	104.51 ± 0.59	87.50	101.87 ± 0.23
Freeze-thaw stability						
LQC	24.00	100.18 ± 3.12	30.00	100.68 ± 4.37	30.00	109.44 ± 2.80
HQC	87.50	110.41 ± 0.63	70.00	105.64 ± 1.56	87.50	103.57 ± 0.94

*Average of three determinations.

### Dilution integrity

The calculated recoveries percent for both dilutions proved that the method was accurate towards the dilution factor as shown in (Table 4).

**Table 4:** Dilution integrity results of AZT, FP and OXY in nasal mucosa matrix.

AZT recovery*%		FP recovery*%	OXY recovery*%
For 25.0% level	97.84	97.23	99.10
For 50.0% level	97.56	98.30	98.22

*Average of three determinations.

### Robustness

Low %RSD was established after a small change in the method flow rate and mobile phase composition (Table 5).

**Table 5:** Robustness results of HPLC determination of AZT, FP and OXY in nasal mucosa.

Parameters	AZT	FP	OXY
Flow rate (± 0.10 mL/min) %RSD*	0.23	0.61	0.53
Organic strength (± 2%) %RSD*	0.17	0.20	0.15

*Average of three determinations.

### Matrix effect

% ME was found to be 55.90, 80.01, and 161.56% for AZT, FP, and OXY, respectively. The results obtained mean that components in the matrix cause suppression effects on AZT and FP, but enhancement effects on OXY.

### System suitability

To ensure that the instrumental system is operating as intended, system suitability testing was carried out (Table 6**)**. Evidently, the parameters fall between the acceptability limits^29^, indicating that the new HPLC technique has demonstrated strong validity and reliability in addition to successfully separating the analytes under study.

**Table 6:** System suitability parameters of the proposed HPLC method for the determination of AZT, FP and OXY in nasal mucosa.

Parameters	Values			Reference
	AZT	FP	OXY	
Retention time (min)	6.61	9.81	2.45	
Capacity factor (k^,^)	7.92	12.16	2.21	K’ > 2.00
Tailing factor (T)	0.97	0.97	1.08	T ≤ 2.00
Number of theoretical plates (N)	8033	3596	5440	*N* > 2000
Height equivalent to theoretical plates (HETP)	0.03	0.06	0.04	The smaller the value, the higher the column efficiency
Selectivity	3.58	1.53	-	ɑ > 1.00
Resolution (RS)	-	20	7.2	RS > 1.50

## Discussions

The purpose of our method is to develop a simple and rapid RP-HPLC-DAD method used for the determination of three co-administrated drugs used for the treatment of seasonal allergy and COVID-19 in the nasal mucosa. To our knowledge, this is the first time for the determination of those drugs in the nasal mucosa. Moreover, the HPLC-DAD method has low cost, which enables us to use our method in routine work.

### Optimization of the chromatographic conditions

The major objective of mobile phase optimization was to obtain good separation with acceptable resolution, peak shape, and sensitivity. The hydrophobicity (Log p) of AZT, FP, and OXY are similar, therefore, their separation was essential. Several mobile phases were investigated by adjusting the composition and pH. Numerous ratios of mobile phases such as acetonitrile: water, methanol: water, and methanol: acetonitrile: water, were tested in isocratic and gradient modes. Likewise, several pH ranges and ratios of buffers were tested. Applying buffer showed no effect on separation, as it gave the same results as water. Finally, isocratic elution with a 70:30 acetonitrile: water ratio and a 0.70 mL/min flow rate was determined to be appropriate for the separation of analytes with a resolution (Rs) > 1.50 and a short runtime.

### Bio-analytical method validation

The FDA’s requirements for bio-analytical validation of the optimized method were followed, and all parameters satisfied the acceptance criteria. For all three proposed analytes, the calibration curve was linear with acceptable accuracy and precision in the ranges of (8-125), (10–100), and (10–125) µg/mL for AZT, FP, and OXY, respectively. The nasal mucosa matrix was found to have a suppression effect on AZT and FP and an enhancement effect on OXY, when the matrix effect on the analytes was also examined.

### Applications for the proposed method

For spiked nasal mucosa, the validated method was used for the determination of three proposed analytes in nasal mucosa with good recovery, as shown in (Table 7**)**.

**Table 7:** Recovery results of AZT, FP and OXY determination by the proposed HPLC method in spiked human nasal mucosa.

Samples	AZT			FP			OXY		
	Spiked conc (µg/mL)	Found conc* (µg/mL)	Recovery %	Spiked conc (µg/mL)	Found conc* (µg/mL)	Recovery %	Spiked conc (µg/mL)	Found conc* (µg/mL)	Recovery%
LQC	24.00	24.47	101.98	30.00	30.90	102.99	30.00	32.95	109.83
MQC	62.50	63.26	101.22	50.00	50.73	101.45	62.50	63.75	102.00
HQC	87.50	88.80	101.49	70.00	72.03	102.90	87.50	89.24	101.99
Mean ± RSD	101.56 ± 0.39			102.45 ± 0.86			104.61 ± 4.52		

*Average of three determinations.

**Fig. 3:**
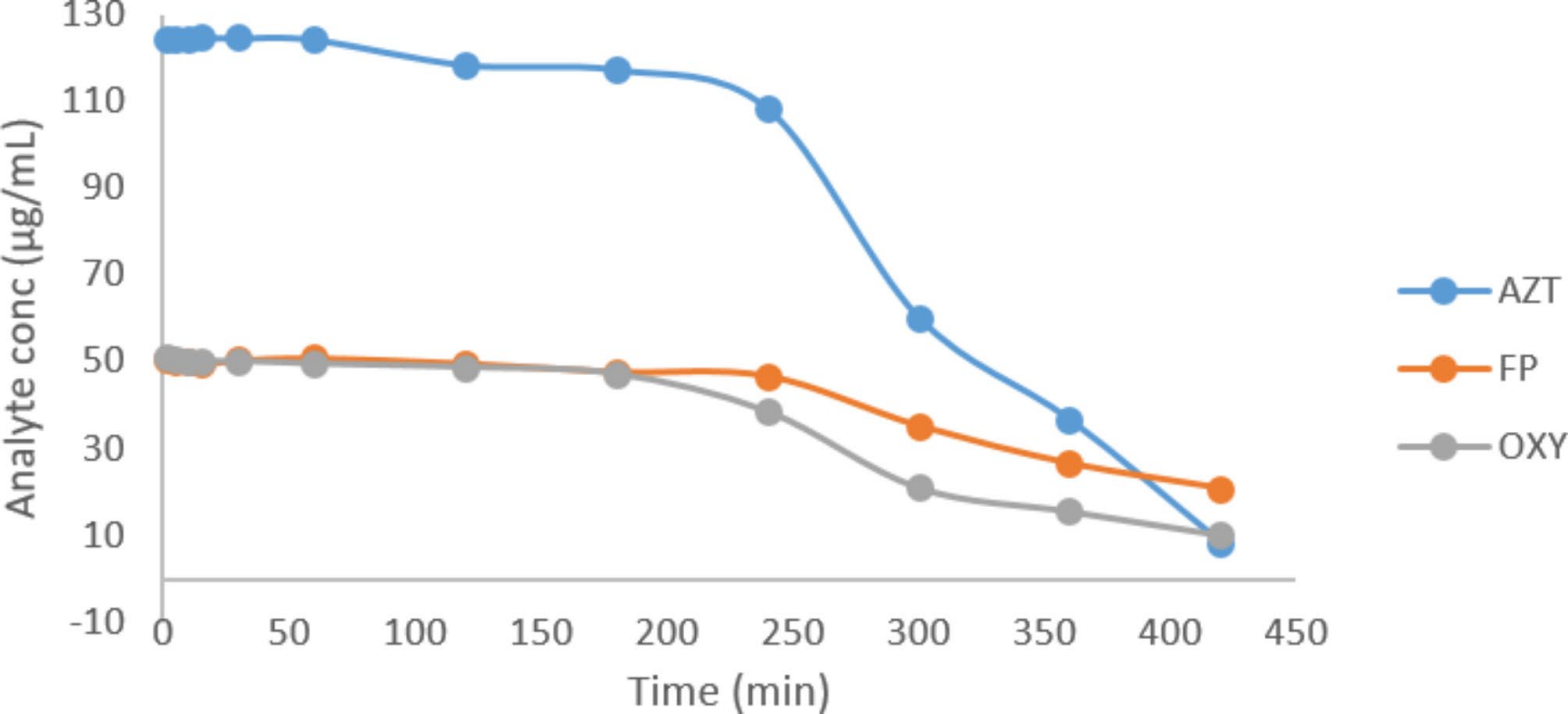
AZT, FP and OXY concentration in nasopharyngeal swabs after single dose administration of azelast nasal spray and oxymet nasal drops.

For real samples, we found that both gender and age do not affect the results of swabs, as they showed good recovery with a minimum % RSD (Table 8). Also, analytes concentration in nasopharyngeal swabs versus time profile after administration of a single dose containing 125, 50, and 50 µg/mL of AZT, FP, and OXY; respectively, were determined, as shown in Fig. 3**and** Table 9. Results showed that the concentration of three proposed analytes is stable in the nose with accepted % RSD for 4 h, then the concentration decreased.

**Table 8:** Recovery results of AZT, FP and OXY determination by the proposed HPLC method in male and female with different ages in nasopharyngeal swabs after single dose administration of azelast plus nasal spray and oxymet nasal drops.

Samples	AZT			FP			OXY		
	Claimed conc (µg/mL)	Found *conc (µg/mL)	Recovery%	Claimed conc (µg/mL)	Found *conc (µg/mL)	Recovery%	Claimed conc (µg/mL)	Found *conc (µg/mL)	Rec.%
Female	125	124.45	99.56	50	50.34	100.68	50	49.77	99.54
Male	125	123.13	98.50	50	49.68	99.35	50	51.17	102.34
Mean ± RSD	99.03 ± 0.75			100.02 ± 0.94			100.94 ± 1.98		
20 years old	125	126.55	101.24	50	49.72	99.44	50	50.74	101.48
40 years old	125	125.90	100.72	50	50.65	101.31	50	49.30	98.59
60 years old	125	124.23	99.38	50	50.66	101.32	50	50.45	100.90
Mean ± RSD	100.45 ± 0.96			100.69 ± 1.08			100.32 ± 1.53		

*Average of three determinations.

**Table 9:** Nasopharyngeal swabs of real samples obtained at different interval times (results were expressed as mean concentration in swab± %RSD).

Time (hours)	AZT		FP		OXY	
	Average concentration in nasopharyngeal swabs (µg/mL)	% RSD	Average concentration nasopharyngeal swabs (µg/mL)	%RSD	Average concentration nasopharyngeal swabs (µg/mL)	%RSD
Immediately	126.55	4.55	49.72	1.20	50.74	1.65
1	123.89	2.18	50.76	7.25	49.18	4.56
2	117.43	1.78	49.71	5.15	49.18	1.00
3	115.01	3.46	47.87	9.49	48.17	5.49
4	104.35	3.51	46.20	1.70	46.74	9.19
5	61.86	5.30	34.48	2.29	20.67	7.58
6	33.69	7.97	26.84	7.01	16.70	5.60
7	8.62	9.97	21.55	8.96	10.70	4.30

## Conclusions

Novel, accurate, and precise isocratic RP-HPLC-DAD method was developed for the simultaneous measurement of AZT, FP, and OXY in nasal mucosa. Distinct calibration curves were created for each of the three target analytes in the nasal mucosa. The FDA requirements were followed in the bio-analytical validation of the procedure. After validation of our method, we applied it to real healthy volunteers after administration of azelast plus nasal spray and oxymet nasal drop. Nasopharyngeal swabs obtained showed the effect of different genders, ages, and times on the concentration of each analyte.

## Data Availability

The datasets used and/or analyzed during the current study available from the corresponding author (Name: Marwa Hassan Mohamed, Gmail: marwahasan.mohamed@pharma.asu.edu.eg ) on reasonable request.
